# Remote delivery of a weight management intervention for adults with intellectual disabilities: Results from a randomized non-inferiority trial

**DOI:** 10.1016/j.dhjo.2024.101587

**Published:** 2024-01-22

**Authors:** L.T. Ptomey, R.A. Washburn, J.R. Sherman, M.S. Mayo, R. Krebill, A.N. Szabo-Reed, J. J. Honas, B.C. Helsel, A. Bodde, J.E. Donnelly

**Affiliations:** a Department of Internal Medicine, The University of Kansas Medical Center, 3901 Rainbow Boulevard, Kansas City, KS, 66160, USA; b Department of Biostatistics & Data Science, The University of Kansas Medical Center, 3901 Rainbow Boulevard, Kansas City, KS, 66160, USA; c Department of Neurology, The University of Kansas Medical Center, 3901 Rainbow Boulevard, Kansas City, KS, 66160, USA

**Keywords:** Down syndrome, Weight loss, Weight maintenance, Diet, Technology

## Abstract

**Background:**

Remote delivery of multi-component weight management interventions results in clinically meaningful weight loss in adults without intellectual disabilities (ID), but the effectiveness of remotely delivered weight management interventions in adults with ID has not previously been evaluated.

**Objective:**

To determine if a weight management intervention delivered remotely could achieve weight loss (kg) at 6 months that is non-inferior to in-person visits in adults with ID and overweight or obesity (BMI ≥25 kg/m^2^).

**Methods:**

Participants were randomized to a 24-mo. trial (6 mos weight loss, 12 mos weight maintenance, 6 mos. no-contact follow up) to compare weight loss achieved with the same multicomponent intervention delivered to individual participants in their home either remotely (RD) or during face-to-face home visits (FTF).

**Results:**

One hundred twenty adults with ID (~32 years of age, 53 % females) were randomized to the RD (n = 60) or the FTF arm (n = 60). Six-month weight loss in the RD arm (−4.9 ± 7.8 kg) was superior to 6-month weight loss achieved in the FTF arm (−2.1 ± 6.7 kg, *p* = 0.047). However, this may be partially attributed to the COVID-19 pandemic, since weight loss in the FTF arm was greater in participants who completed the intervention entirely pre-COVID (n = 33,−3.2 %) compared to post-COVID (n = 22, −0.61 %). Weight loss across did not differ significantly between intervention arms at 18 (p = 0.33) or 24 months (p = 0.34).

**Conclusion:**

Our results suggest that remote delivery is a viable option for achieving clinically relevant weight loss and maintenance in adults with ID.

**Nct registration:**

NCT03291509.

## 1. Introduction

An intellectual disability (ID) is defined as a developmental disability identified prior to age 22 characterized by significant limitations in both intellectual functioning (IQ ≤ 75) and adaptive behaviors, including conceptual, social, interpersonal, and practical skills.^1^ Adults with ID represent ~1–3 % of the US population.^2^ The prevalence of obesity (BMI ≥30 kg/m^2^) and obesity-associated health conditions including cardiovascular disease,^3,4^ hypertension,^5^ type 2 diabetes,^6^ and obstructive sleep apnea,^5^ are higher in adults with ID compared with adults without ID. Low levels of physical activity (PA), high sedentary behavior,^7^ unhealthy diet,^8,9^ food insecurity,^10^ challenges with transportation,^11^ limited control over health behaviors,^12^ genetic predisposition,^13^ the use of obesogenic medications,^14–16^ and living arrangements are thought to contribute to obesity in adults with ID.

Although limited, research by our group^17,18^ and others^19–22^ has demonstrated that multicomponent weight management interventions adapted for use in adults with ID can produce clinically meaningful weight loss of ≥5 %, and successful weight maintenance. These successful weight management interventions were all conducted in accordance with current weight management guidelines,^23^ and were delivered using individual in-person home visits,^17–19,21,22^ or through a community occupational day center.^20^ Although successful, both provider and participant cost as well as logistical burden (e.g. access to transportation, time associated with travel) associated with these delivery strategies, specifically individual in-person home visits, greatly limits the reach, adoption, and implementation of these interventions by clinic programs and community agencies serving adults with ID. Remote delivery of multi-component weight management intervention, e.g., phone, Zoom^®^ etc., which eliminates travel associated with in-person home visits, has been shown to result in clinically meaningful weight loss and maintenance in adults without ID.^23–25^ However, the effectiveness of remote delivery for weight loss and maintenance in adults with ID has not been previously evaluated. Thus, we conducted a randomized trial in adults with ID to determine if a weight management intervention (6 months weight loss, 12 months maintenance, 6 months no contact follow up) delivered remotely could achieve clinically meaningful weight loss (kg) at 6 months that is non-inferior to weight loss achieved with the same intervention delivered using individual in-person home visits.

## 2. Methods

### 2.1. Overview

We randomized 120 adults with mild-to-moderate ID and overweight or obesity (BMI ≥25 kg/m^2^) to a 24-month weight management trial, consisting of 6 months of weight loss, 12 months of weight maintenance, and 6 months no-contact follow up. Participants were asked to follow the same multicomponent weight management intervention and were randomized either remote delivery (RD) or face-to face delivery (FTF). The RD group included monthly health education sessions conducted over video conferencing (Zoom^®^ Video Conferencing Inc. San Jose, CA) and self-monitoring of diet (Lose it! App, Fitnow Inc., Boston, MA), physical activity (Fitbit^®^, Google LLC, Mountain View, CA) and weight (Fitbit^®^, Google LLC, Mountain View, CA) on a tablet computer (iPad^®^, Apple Inc., Cupertino, CA). The FTF group included monthly health education sessions conducted at the participants home, self-monitoring of diet and PA using paper tracking sheets, and the use of a standard scale for self-monitoring of weight. The primary aim was to determine if 6-month weight loss (kg) using RD was non-inferior to weight loss achieved using FTF delivery. This trial, which was approved by the Institutional Review Board and registered on clinicaltrials.gov (NCT03291509) was conducted in the United States from March 2018 to September 2022. The study design and procedures were guided by our community advisory board consisting of adults with ID, caregivers, and direct service professionals. A detailed description of the rationale, design, participant eligibility criteria, and methods for this trial have been previously published,^26^ below we provide a brief summary of the methods as well as protocol deviations that occurred due to the COVID-19 pandemic.

### 2.2. Participant eligibility

*Inclusion criteria*: *1)* Age 18 yrs and older with a diagnosis of mild to moderate ID obtained from Community Developmental Disability Organizations records. *2)* BMI of 25–45 kg/m^2^. Individuals with a BMI <25 kg/m^2^ are not overweight, and individuals with a BMI >45 kg/m^2^ require more aggressive intervention (e.g., surgery, medication, etc.). *3)* Sufficient functional ability to understand directions, communicate preferences, wants, and needs through spoken language, as strategies necessary for intervention delivery are likely to differ from those used with individuals who are unable to communicate through spoken language. *4)* Living at home with a parent/guardian, or in a supported living environment with a caregiver who assists with food shopping, meal planning, and meal preparation and agrees to serve as a study partner. *5)* No plans to relocate outside the study area over the next 24 mos. *6)* Internet access in the home. *Exclusion criteria. 1)* Unable to participate in MVPA as assessed by physician’s consent. *2)* Insulin dependent diabetes as this condition requires medical monitoring beyond the scope of this study. *3)* Participation in a weight management program involving diet and PA in the past 6 mos. *4)* Serious food allergies that would prevent them from following the prescribed diet 5) Diagnosis of Prader-Willi Syndrome, as diet strategies needed for successful weight management are likely to differ from those with other IDs. *5)* Pregnancy. *6)* Serious medical risk, e.g., cancer, recent heart attack, stroke, angioplasty as determined by the PCP. *7)* Unwilling to be randomized.

### 2.3. Recruitment/randomization

Participants were recruited through community organizations as well as social media and word of mouth. Written informed consent was obtained from either the participant (self as guardian) or a legal guardian with participant assent, as well as the caregiver/study partner. Participants were randomized, stratified by the number of participants in a residence (1 or >1). Treatment allocation sequences were generated by computer software using block randomization with equal allocation to the RD and FTF arms. Allocation was concealed in envelopes and revealed to the study coordinator as participants were recruited.

### 2.4. Study partner role

Participants were asked to select a parent/guardian, residential support staff, or an individual who assists with food shopping/meal preparation to serve as a study partner for each participant. Study partners were asked to attend all monthly behavioral education sessions, and to support, encourage, and assist participants in complying with the study protocol.

### 2.5. Intervention components

In accordance with current American Heart Association, American College of Cardiology, and the Obesity Society (AHA/ACC/TOS) guidelines for the management of overweight and obesity in adults,^28^ all participants followed a multicomponent weight loss/maintenance intervention which included an enhanced stop light diet, increased PA, self-monitoring of diet and PA, and behavioral education/counseling. Participants in RD arm were provided with iPads^®^, which were pre-loaded with the Zoom^®^ video conferencing software, the Lose It! app for self-monitoring diet (Fitnow Inc., Boston, MA) and the Fitbit^®^ (Google LLC, Mountain View, CA) app for self-monitoring PA and weight. Participants in the FTF arm were given paper tracking sheets and a pedometer (Omron HJ-320, Lake Forest, IL.) for self-monitoring diet and PA. Participants were assigned to an interventionist for the duration of the study.

### 2.6. Diet

Participants in both intervention arms were prescribed an enhanced stop light diet (eSLD). The eSLD utilizes the stop light diet^27^ enhanced by recommending the consumption of high volume, low energy portion-controlled entrées and shakes, and fruits and vegetables. During weight loss (0–6 months) participants were encouraged to consume a minimum of 2 entrées (200–300 kcals each), 2 shakes (~100 kcal each), 5 one-cup servings of fruits and vegetables each day, and other lower energy foods (green/yellow) from a chart/picture of foods that were color-coded based on the SLD system to achieve an energy intake of 1200–1500 kcal/d for women and 1500–1800 kcal/d for men.^23^ Participants were asked to purchase portion-controlled entrées from a list of over 100 entrées ($1–$4 each) that satisfied study requirements for energy content. The recommended low energy shakes (Profile by Sanford Health, Sioux Falls, SD) were provided by the trial during weight loss and were shipped to the participant’s homes monthly. During both weight maintenance (7–18 months) and the no-contact follow-up (19–24 months) participants were encouraged to consume portion-controlled entrées, 5 one-cup servings of fruits/vegetables each day and use the SLD system to make daily food choices.

### 2.7. Physical activity

Participants in both intervention arms were asked to complete 150 min/wk of moderate to vigorous intensity PA (≥3 METs) as recommended for all adults by the American College of Sports Medicine^28^ and the U.S. Department of Health and Human Services.^29^ Suggested MVPA included walking, cycling, swimming, active video games, no-cost online workouts, and active recreational sports (soccer, tennis, basketball, etc.). All participants were provided the Fit 5 Fitness Guide and videos created by the Special Olympics.^30^

### 2.8. Behavioral education

Participants and study partners in both intervention arms were asked to attend monthly ~30–45-min behavioral education sessions with an interventionist across the 18-month active intervention. Interventionists were randomly assigned to participants in both intervention arms to diminish the potential for interventionist bias. These sessions were identical in content and were delivered using Zoom^®^ or during in-person home visits for the RD and FTF arms, respectively. During each session interventionists provided feedback on compliance with the intervention, answered questions, problem-solved, provided support for achieving both diet and PA recommendations, and reviewed a short lesson on different topics related to weight management. *COVID-19 protocol deviation FTF arm*. COVID restrictions prohibited FTF contacts with participants from March through June 2020, thus all sessions with participants/study partners in the FTF arm were conducted by audio only telephone during this period (n = 37). Following the lifting of COVID restrictions 13 participant/study partner dyads in the FTF arm were uncomfortable with resuming FTF visits and were allowed to continue behavior sessions by phone from July 2020 through the completion of the active intervention in February 2022. There were no disruptions in the delivery of the weight loss shakes during this time.

### 2.9. Self-monitoring

Participants in both intervention arms were asked to self-monitor diet, PA, and body weight across the 24-month trial. All self-monitoring data was available to interventionists for participant counseling during behavioral education sessions. Participants in the RD arm were asked to self-monitor diet using the Lose It! App on the iPad^®^, wear a Fitbit^®^ Charge 2 Activity Tracker on the non-dominant wrist to monitor daily PA, and weigh on a Fitbit^®^ Aria digital scale during each behavioral education session. Diet, PA, and weight data were accessible to the interventionist through the apps to inform participant counseling during the monthly behavioral education sessions.

Participants in the FTF arm were asked to self-monitor diet using a paper tracking sheet containing pictorial representations of each food category and record the minutes of PA and the number of pedometer steps each day. These tracking sheets were collected by the interventionist during monthly home visits and were used to inform participant counseling. Participant weight was obtained during each behavioral education session using a calibrated digital scale (Belfour Model PS6600, Saukville, WI). *COVID-19 protocol deviation FTF arm:* Participant self-report data for diet, PA, and weight was discussed during the phone call rather than during an in-person review of participant paper records by interventionists. Monitoring of body weight was not possible using phone delivery.

### 2.10. Incentives

Participants in both intervention arms received a $5.00 gift card for each behavioral session attended, $20.00 for completing each of the 5 outcome assessments, and both groups were given an iPad^®^ on study completion (18 months). Study partners received $50.00 gift cards at 6, 12, and 18 months to compensate for the additional burden associated with participation in this trial.

## 3. Outcomes

Anthropometrics, i.e., weight, height and waist circumference were assessed by research staff blind to intervention arm during ~30-min home visits at baseline, 6, 12, 18 and 24 months. Process outcomes were assessed on the following schedule: Adherence with diet and PA recommendations was assessed at 6, 12 and 18 months. Behavioral session attendance and adherence with self-monitoring of diet and PA were assessed across the 18-month intervention.

### 3.1. Anthropometrics

Participants were weighed to the nearest 0.25 kg, on a calibrated scale (Model PS6600, Belfour, Saukville, WI) between 8 and 10 a.m. while wearing shorts and a t-shirt following a minimum 12-h fast. Standing height was measured with a portable stadiometer (Model IP0955, Invicta Plastics Limited, Leicester, UK). BMI was calculated as weight (kg)/height (m^2^). Waist circumference, as a surrogate for abdominal adiposity, was assessed using the procedures described by Lohman et al.^31^ Three measurements were obtained with the outcome recorded as the average of the closest 2 measures. *COVID-19 protocol deviation-body weight assessment*. Body weight was assessed by delivering the calibrated digital scale to participant’s homes in a box. Staff phoned participants and directed them to retrieve and set-up the scale on a firm, non-carpeted, surface in their home. Participants were asked to step on the scale and take a cell-phone photo of the digital display and text the photo to the research staff. We were unable to develop workable protocols for no contact assessments of waist circumference and height which resulted in missing data for waist circumference and BMI (kg/m^2^) for 20, 12, and 14 participants at 6, 18, and 24 months, respectively.

### 3.2. Process outcomes

Behavioral session attendance was obtained from interventionists records and expressed as the percentage of all scheduled sessions attended.

Adherence with diet recommendations was estimated as the mean number of shakes and entrées consumed per day assessed by image-assisted 3 day food records (2 wk days/1 wk end day) collected at 6 and 18 month.^32^

Adherence with self-monitoring of diet and PA was assessed from interventionists records from monthly behavioral sessions and expressed as the percentage of prescribed monitoring days completed.

### 3.3. Statistical power and analysis

#### Power.

Our primary aim was to determine whether weight loss across 6 months using RD was non-inferior to (as good as) weight loss observed using the same intervention delivered FTF. We chose a non-inferiority margin of 3 kg based on results from our previous trial in adults with ID using FTF delivery which elicited 6-month weight loss of −6.8 ± 5.5 kg. The 3 kg margin represents the maximal between group difference that would yield clinically meaningful weight loss in the RD arm.^18^ Power analysis indicated that randomization of 120 participants with equal allociation to the RD and FTF arms, and accounting for 20 % attrition, would provide 84 % power to demonstrate non-inferiority assuming the 3 kg margin for the between arm difference in weight loss (kg) and a standard deviation of 5.5 kg.

#### Missing data.

Analysis of our primary aim was based on both intent-to-treat principles using traditional multiple imputation for missing weight data and a completers only analysis.^33^ Missing weights at 6 months were not related to intervention arm or participant demographic characteristics; therefore, Monte Carlo Markov Chain multiple imputation^34^ (SAS Proc MI, k = 10) was used to impute missing 6 month weight. Imputation was not used for any other outcomes or at the 18 or 24 month periods.

#### Analysis-primary aim.

We constructed a two-sided 95 % confidence interval (CI) for the difference in mean 6 mo weight loss (kg) between the FTF and RD arms (FTF minus RD). Non-inferiority for 6 month weight loss in the RD arm would be demonstrated if the lower bound of the 95 % CI for the between arm difference in weight loss (FTF minus RD) is > −3 kg and the upper limit is > 0 kg. Pre-specified criteria for demonstrating superiority of 6 month weight loss (kg) in the RD vs. the FTF arm were a lower limit of the 95 % CI around the between arm difference in weight loss (FTF-RD) >0 kg and an upper limit of >4 kg.

### 3.4. Additional analyses. All additional analysis, based on superiority hypotheses, were unpowered

Changes in weight (kg, %), BMI and waist circumference across 6, 18 and 24 months were examined independently using two sided two-sample t-tests between the RD and FTF arms. Linear mixed modeling assuming an autoregressive correlation structure over time was used to compare changes in mean weight loss (kg and %) between the RD and FTF arms across 24 months. Separate two sample t-tests were used to compare intervention adherence (behavioral session attendance and adherence to dietary, PA and self-monitoring recommendations) between the RD and FTF arms across 6 and 18 months. Linear mixed models were used to examine the association of participant characteristics (age, sex, minority status, Down syndrome diagnosis, living situation) and intervention adherence (behavioral session attendance and adherence to dietary, PA and self-monitoring recommendations) with weight change across 18 months. Statistical procedures were performed using SAS version 9.4.

## 4. Results

### 4.1. Participants

**Fig. 1.**
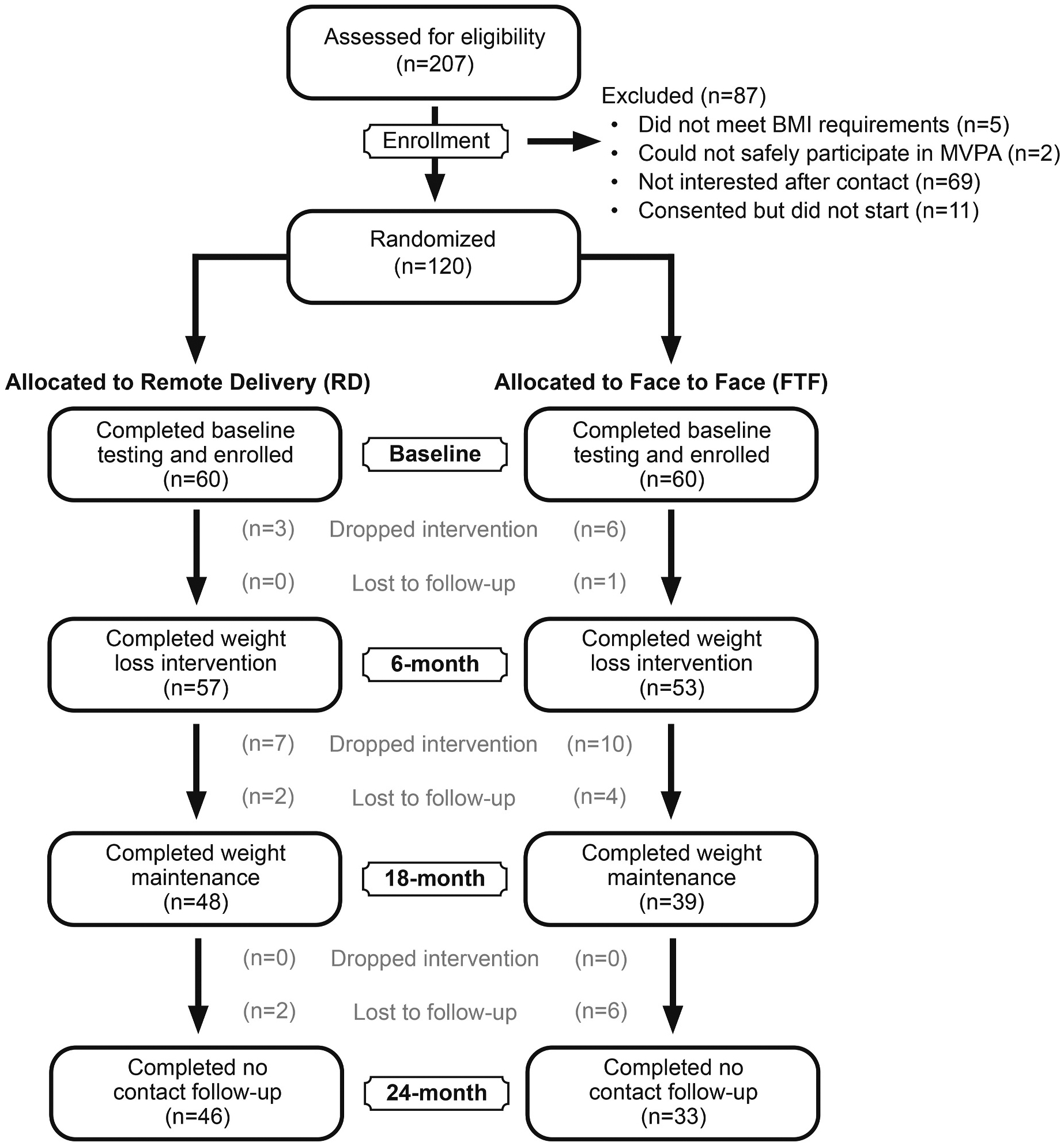
Consort diagram.

One hundred twenty adults with ID were randomized to the FTF (n = 60) or the RD (n = 60) arm. Body weight was obtained from 53 (88 %), 39 (65 %) and 33 (55 %) participants in the FTF arm at 6, 18 and 24 months, respectively and 57 (95 %), 48 (80 %) and 46 (77 %) participants in the RD at the same time periods (Fig. 1). Participant retention was lower in the FTF compared with the RD arm at 6 (FTF: 88 %, RD: 95 %), 18 (FTF: 65 %, RD: 80 %) and 24 months (FTF: 55 %, RD: 77 %). There were no significant differences (all p > 0.05) in age (32. vs. 31 years), sex (53 vs. 46 % female), minority status (17 % vs. 7 % minority) and living situation (56 % vs. 41 % living at home) between participants who completed vs. those who did not complete the intervention. No serious adverse events related to the intervention occurred during the 18-month intervention or the 6-month no contact period. Baseline participant characteristics are presented in Table 1. Participants were ~32 years of age, 53 % females, 78 % non-Hispanic white.

## 5. Primary outcome

**Fig. 2.**
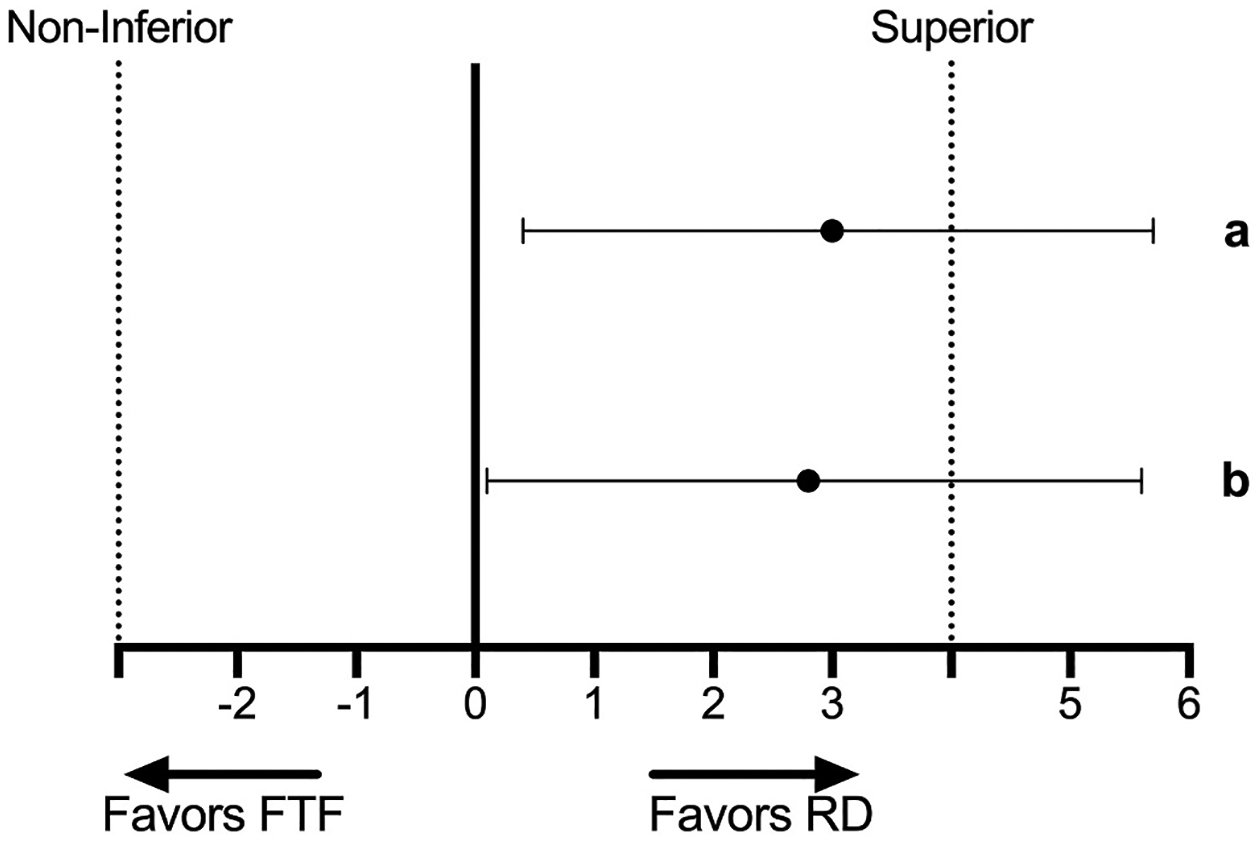
Difference in Weight Loss (kg) (Face to Face minus Remote Delivery) ± 95 % confidence intervals for both (a) imputed data and (b) completers only.

Fig. 2 illustrates the mean difference in 6-month weight loss between the FTF and RD arms (kg) with the two-sided 95 % CI for both the intent-to-treat and completers only analyses. The between arm difference and 95 % CI for weight change (FTF minus RD) was 2.8 kg (95 % CI 0.04 to 5.6) (*p* = 0.047) for completers only and 2.9 kg (95 % CI 0.3 to 5.4) (*p* = 0.030) using imputation for missing weight change data. The lower limit of the 95 % CI around the difference in weight loss between the FTF and RD arms was >0 and the upper limit was >4 kg for both the intent-to-treat and completer analysis, indicating 6-month weight loss in the RD arm was superior, thus also non-inferior, to 6-month weight loss achieved in the FTF arm. Weight loss in the FTF arm was greater in participants who completed the 6 month intervention entirely in-person (before March 2020) (n = 33,−3.2 %) compared to participants completed the intervention post-COVID which included a mixture of in-person and phone visits (n = 22, −0.61 %). However, 6-month weight loss in the RD arm was similar in participants who completed the intervention pre (n = 34, −5.5 %) and post-COVID (n = 23, −4.4 %).

### 5.1. Additional analyses/outcomes

**Fig. 3.**
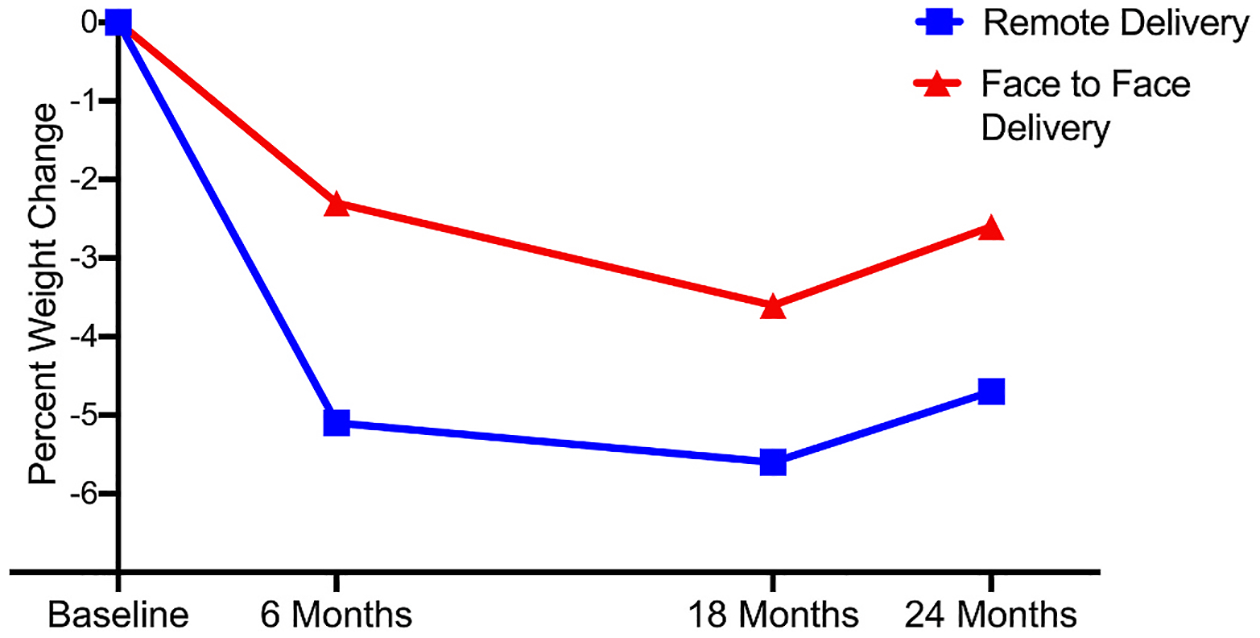
Percent change in weight in adults with intellectual disabilities across 24-months using a completers only analysis.

Changes in weight, BMI and waist circumference following the weight loss (0–6 mos) and weight maintenance interventions (0–18 mos) and across the entire 24-month trial for completers only are presented in Fig. 3 and Table 2.

#### Weight loss intervention (0–6 months).

The decrease in BMI was significantly greater in the RD compared with the FTF arm (*p* = 0.046); however, the decrease in waist circumference in the RD and FTF arms did not differ significantly (*p* = 0.16).

#### Weight loss + maintenance (0–18 months).

Weight loss and the decrease in both BMI and waist circumference did not differ significantly by intervention arm (all *p* > 0.05).

#### Weight loss + maintenance + no-contact follow up (0–24 mos).

Weight loss and the decrease in both BMI and waist circumference did not differ significantly by intervention arm (all *p* > 0.05). Longitudinal modeling of weight change over 24-monhs controlling for baseline weight indicated no significant difference between intervention arms (*p* = 0.83).

### 5.2. Intervention adherence

Behavioral session attendance, consumption of portion-controlled shakes and entrées, and percent of study days participants self-monitored diet and PA is presented in Table 3. We observed no significant differences in behavioral session attendance(*p* = 0.67) or in self-monitoring of diet (*p* = 0.44) between the RD and FTF arms across the 18-month study. However, self-monitoring of PA was significantly higher in the RD (58 %) compared with the FTF arm (40 %, *p* = 0.006). There were no significant differences between the RD and FTF arms in servings of portion-controlled shakes and entrées at both 6 and 18 months (all *p* > 0.05).

### 5.3. Factors impacting weight loss

Age, sex, minority status, Down syndrome diagnosis, and living situation were not associated with weight loss at 6, 18, or 24 months (all *p* > 0.05). Attendance at both monthly behavioral education sessions (r = 0.29, *p* = 0.006) was associated with greater weight loss across 18 months. Self-monitoring of diet and PA were not associated with weight loss across 18 months (both *p* > 0.05).

## 6. Discussion

The results of the current trial suggest that remote delivery of a multicomponent weight management intervention to individual adults with ID and a study partner achieves 6-month weight loss that is superior to weight loss achieved with the identical intervention delivered FTF. No significant between arm differences in weight loss following completion of a weight maintenance intervention (18 mos) or following 6-month no contact period were observed. However, this may be partially attributed to high attrition at 18 and 24 months in the FTF group. The clinically relevant 6-month weight loss observed in the RD arm was maintained at both the completion of a 12-month maintenance intervention and following a 6-month no contact period. In total, our results suggest that remote delivery of the multicomponent intervention evaluated in this trial represents a viable option for achieving clinically relevant weight loss and maintenance in adults with ID.

The observation that 6-month weight loss in the RD arm was superior to the FTF arm was unexpected and inconsistent with results from previous trials in both individuals without ID and individuals with ID.^35,36^ Additionally, previous weight management trials in adults without ID by our group^24,37^ and others^38–40^ have observed similar weight loss using interventions delivered remotely or in-person. The superiority for 6-month weight loss in the RD arm was likely a function of the less than expected 6-month weight loss in the FTF arm (−6.8 kg, expected, −2.1 kg observed) which may be at least partially attributed to the COVID-19 pandemic which appeared to have a greater impact on weight loss and participant retention in the FTF arm compared with the RD arm, most likely due to deviations in the study protocol in the FTF arm to accommodate COVID restrictions on in-person contact. Thus, the finding of superiority of 6-month weight loss in the RD over the FTF arm should be cautiously interpreted.

The clinically relevant mean weight loss/maintenance observed in the RD arm in the current trial was encouraging, as participants were able to maintain weight loss across 24 months, including a 6 month no-contact period. These results are similar to the results from our previous 18 month multi-component weight management trial in 150 adults with ID which used an eSLD and FTF home visits where mean weight loss was −7.0 % and −6.7 % at 6 and 18 months, respectively.^18^ The magnitude of weight loss in the RD arm in the current trial and previous trials using the eSLD in adults with ID by our group^17,18^ exceeds the magnitude of weight loss reported in the limited number of previous trials using conventional reduced energy meal plan diets in adults with ID, which report mean weight loss ranging from −3.3 % to −4.4 % at 6 months^19,21^ to −3.8 % to −4.4 % at 12 months.^21,22^

Poor reported compliance with the dietary, PA, and self-monitoring recommendations observed in this trial are discordant with the magnitude of the observed weight loss in the RD arm at both 6 (−5.1 %) and 18 months (−5.6 %). Previous multi-component weight management trials in adults with ID by our group^17,18^ and others^19,21,22^ have also observed clinically relevant weight loss despite poor compliance with dietary, PA and self-monitoring recommendations. These observations highlight the difficulty in assessing dietary intake and PA frequently noted in adults with ID^18,41–43^ and calls into question the effectiveness of recommending increased PA and self-monitoring of diet and PA in multi-component weight management interventions in adults with ID, topics worthy of further investigation.

While not an aim of this study, several aspects of the RD intervention suggest the potential for this approach to improve the reach, adoption, implementation, and maintenance of weight management for adults with ID and overweight and obesity. From the provider perspective, remote delivery eliminates the time and expense associated with staff travel to conduct FTF home-visits which allows for the delivery of weight management to adults with ID regardless of their geographic location. Additionally, our approach requires minimal caregiver/study partner involvement, i.e., attendance at monthly behavioral education sessions and providing support, encouragement, and assistance to participants in complying with intervention protocol and a reasonable frequency of participant/study partner contact, i.e., monthly 30 to 45-min behavioral education sessions over 18 months. Future research is needed to examine if the RD intervention can be successfully implemented into clinical practice.

Strengths of this trial include a randomized design that was adequately powered to evaluate the primary aim, inclusion of a 6 month no-contact follow-up after completion of the 18-month active intervention, the use of an intervention tailored to the cognitive abilities of adults with ID, assignment of the same interventionist to pairs of participants (RD, FTF) to reduce the potential for interventionist bias, and high compliance with the behavioral session protocol (~79 %). As discussed previously, primary limitation in this trial were the inability to obtain useful assessments of participant compliance with the dietary and PA recommendations and protocol deviations in intervention delivery in the FTF arm necessitated by the COVID-19 pandemic. Finally, this trial was conducted in a sample of adults with mild-to-moderate ID and overweight/obesity who lived in the community and who volunteered, and were incentivized, to participate in a weight management trial. Thus, these results may not be generalizable to adults with more severe ID, those living in institutional settings, or outside of the context of a research trial.

## 7. Conclusion

Our results suggest that remote delivery of the multicomponent intervention evaluated in this trial is a viable option for achieving clinically relevant weight loss and maintenance that has the potential to improve the reach, adoption, implementation, and maintenance of weight management for underserved adults with ID and overweight and obesity.

## Figures and Tables

**Table 1:** Baseline characteristics of adults with intellectual disabilities randomized to either a remotely delivered (RD) or face-to-face (FTF) weight management intervention.

	Intervention arms	
Variable	RD (n = 60)	FTF (n = 60)
Age (yrs.)^a^	30.6 ± 9.0	33.5 ± 11.7
Weight (kg)	94.4 ± 25.8	101.5 ± 27.5
BMI (kg/m^2^)	36.0 ± 7.0	38.8 ± 8.5
Female	28 (46.7 %)	35 (58.3 %)
Minority^b^	10 (16.7 %)	16 (26.7 %)
Diagnosis		
Down Syndrome	17 (28.3 %)	22 (36.7 %)
Autism	15 (25.0 %)	15 (25.0 %)
Other	28 (46.7 %)	23 (38.3 %)
Living Environment		
At home with a parent	34 (56.7 %)	29 (48.3 %)
Alone with Family Support	3 (5.0 %)	3 (5.0 %)
Alone with paid caregiver support	11 (18.3 %)	10 (16.7 %)
In a group setting	12 (20.0 %)	18 (30.0 %)

a Values are mean ± standard deviation or sample size (n) and percent.

b Race/Ethnicity other than non-Hispanic white.

**Table 2:** Changes in weight, BMI, and waist circumference (WC) across 6, 18 and 24 months in adults with intellectual disabilities randomized to a remotely delivered (RD) or face-to-face (FTF) weight management intervention using a completers only analysis.

	Weight Loss *0*–6 months					Weight Loss + Maintenance *0*–18 months					Weight Loss + Maintenance + No contact follow-up *0*–24 months				
	RD		FTF		*p*	RD		FTF		*p*	RD		FTF		*p*
	n	*M* ± SD	n	*M* ± SD		n	*M* ± SD	n	*M* ± SD		n	*M* ± SD	n	*M* ± SD	
Weight (kg)	57	−4.9 ± 7.8	53	−2.1 ± 6.7	0.047	48	−5.4 ± 9.9	39	−3.4 ± 9.0	0.325	46	−4.5 ± 10.3	33	−2.3 ± 10.1	0.340
Weight (%)	57	−5.1 ± 7.7	53	−2.3 ± 6.5	0.047	48	−5.6 ± 9.9	39	−3.6 ± 8.8	0.339	46	−4.7 ± 10.5	33	−2.6 ± 9.1	0.362
BMI (kg/m^2^)	50	−1.9 ± 2.9	40	−0.7 ± 2.6	0.046	40	−1.9 ± 3.9	36	−1.2 ± 3.7	0.407	36	−2.2 ± 4.2	29	−1.2 ± 4.2	0.342
WC (cm)	49	−4.6 ± 7.8	40	−2.3 ± 7.2	0.164	40	−2.0 ± 13.1	36	−2.0 ± 7.9	0.980	36	−4.8 ± 8.4	29	−1.6 ± 9.1	0.150

**Table 3:** Intervention adherence of adults with intellectual disabilities randomized to either a remotely delivered (RD) or face-to-face (FTF) weight management intervention.

	0–6 months			0–18 months		
	RD	FTF	*p*	RD	FTF	*p*
Behavioral session attendance (% of days)	86.9 ± 21.3	84.2 ± 22.6	0.49	79.6 ± 22.6	77.7 ± 22.9	0.67
Portion-controlled shakes (servings/day)^a,c^	1.2 ± 1.5	1.0 ± 1.8	0.15	0.4 ± 1.1	0.7 ± 2.0	0.15
Portion-controlled entrees (servings/day)^b,c^	1.5 ± 1.5	0.9 ± 2.0	0.15	0.5 ± 1.2	0.7 ± 2.0	0.23
Self-monitoring diet (% of days)^d^	57.2 ± 35.4	51.9 ± 34.2	0.44	57.2 ± 35.3	51.9 ± 34.3	0.44
Self-monitoring physical activity (% of days)^e^	65.5 ± 31.4	45.2 ± 38.1	0.002	58.1 ± 32.5	40.1 ± 34.0	0.006

c RD = Days tracked using Fitbit^®^, FTF = Days tracked on paper tracking sheets.

a Recommended during weight loss only.

b RD = Days tracked on Lose It! app, FTF = Days tracked on paper tracking sheets.

c Collected only at 6 and 18 months.
